# American Medical Association

**Published:** 1873-03

**Authors:** Wm. B. Atkinson

**Affiliations:** Permanent Secretary, 1400 Pine Street, Southwest corner of Broad, Philadelphia


### AMERICAN MEDICAL ASSOCIATION.

The twenty-fourth annual session will be held in St. Louis, Missouri, May 6, 1873, at 11 A.M.

The following committees are expected to report:

*On Cultivation of the Cinchona Tree.*—Dr. Lemuel J. Deal, Philadelphia, Pennsylvania, Chairman.

*On Measures to Prevent the Extension of Diseases of Inferior Animals to Man, and the Sanitary Measures to Arrest the Progress of such Diseases in Animals.*—Dr. A. W. Stein, New York, Chairman.

*Owing to the removal from the city of Dr. Ray, Dr. Alexander becomes Chairman.

*On the Treatment of Fractures.*—Dr. Lewis A. Sayre, New York, Chairman.

*On Gunguillia, a Substitute for Quinia.*—Dr. William Chew Van Bibber, Baltimore, Maryland, Chairman.

*On Gynæcology.*—Dr. Montrose A. Pallen, St. Louis, Missouri, Chairman.

*On the Renewal of Prescriptions without Authority, and on the Relations of Physicians and Druggists.*—Dr. R. J. O'Sullivan, New York, Chairman.

*On Vaccination.*—Dr. T. N. Wise, Covington, Kentucky, Chairman.

*On Skin Transplantation.*—Dr. J. Ford Thompson, Washington, D. C., Chairman.

*On Some Diseases Peculiar to Colorado.*—Dr. John Elsner, Denver, Colorado, Chairman.

*On Correspondence with State Medical Societies.*—Dr. N. S. Davis, Chicago, Illinois, Chairman.

*On National Health Council.*—Dr. Thomas M. Logan, Sacramento, California, Chairman.

*On Nomenclature of Diseases.*—Dr. Francis Gurney Smith, Philadelphia, Pennsylvania, Chairman.

*On American Medical Necrology.*—Dr. J. D. Jackson, Danville, Kentucky, Chairman.

*On Suggestions on Medical Education.*—Dr. A. M. Pollock, Pittsburg, Pennsylvania, Chairman.

*On Medical Education.*—Dr. William Carson, Cincinnati, Ohio, Chairman.

*On Medical Literature.*—Dr. Austin Flint, New York, Chairman.

*On Prize Essays.*—Dr. John S. Moore, St. Louis, Missouri, Chairman.

*On Plan for better Arrangement of Sections and more rigid Examination of Papers offered for Publication.*—Dr. E. L. Howard, Baltimore, Maryland, Chairman.

*On Ethics.*—Dr. H. F. Askew, Wilmington, Delaware, Chairman.

Physicians desiring to present papers before the Association should observe the following rule:

"Papers appropriate to the several Sections, in order to secure consideration and action, must be sent to the Secretary of the appropriate Section at least one month before the meeting which is to act upon them. It shall be the duty of the Secretary to whom such papers are sent, to examine them with care, and, with the advice of the Chairman of his Section, to determine the time and order of their presentation, and give due notice of the same.

OFFICERS OF SECTIONS.

*Chemistry and Materia Medica.*—Drs. R. E. Rogers, Philadelphia, Chairman; Ephraim Cutter, Boston, Mass., Secretary.

*Practice of Medicine and Obstetrics.*—Drs. D. A. O'Donnell, Baltimore, Maryland, Chairman; Benjamin F. Dawson, New York City, Secretary.

*Surgery and Anatomy.*—Drs. Edward Warren, Baltimore, Maryland, Chairman; W. F. Peck, Davenport, Iowa, Secretary.

*Meteorology and Epidemics.*—Drs. George Sutton, Aurora, Indiana, Chairman; Elisha Harris, New York City, New York, Secretary.

*Medical Jurisprudence, Hygiene and Physiology.*—Drs. S. C. Busey, Washington City, Chairman; A. B. Arnold, Baltimore, Maryland, Secretary.

*Phychology.*—Drs. Isaac Ray, Philadelphia, Pennsylvania, Chairman; John Curwen, Harrisburg, Pennsylvania, Secretary.

AMENDMENTS TO CONSTITUTION TO BE ACTED ON.

*Resolved*, That the United States Marine Hospital Service be placed in the same relative position in the American Medical Association as the Medical Departments of the United States Army and Navy.

And that, in paragraph two, of the second section, after the words "army and navy," the words "and the United States Marine Hospital" be inserted.

AMENDMENTS TO BY-LAWS TO BE ACTED ON.

*Section III.*—Standing Committees.

That, instead of a report on Medical Education, on Medical Literature, and Climatology and Epidemic Diseases, there shall be annually delivered before the Association, at its general meetings, an address in Medicine, an address in Surgery, and an address in Midwifery, or the Diseases of Children, the lecturer to be appointed this year by the President; afterwards by the Committee on Nominations.

Also, in section six, after the words, "the chiefs of the bureaus of the army and navy," be inserted "and the supervising surgeon of the United States Marine Hospital Service."

Secretaries of all medical organizations are requested to forward lists of their delegates as soon as elected, to the permanent Secretary.

WM. B. ATKINSON, M.D., *Permanent Secretary*,
1400 Pine Street, Southwest corner of Broad, Philadelphia.

